# ESPO AND BEHAVIORAL AND SOCIAL SCIENCES SECTION SYMPOSIUM: BRIDGES TO ELDER JUSTICE: BUILDING COLLABORATIONS AND COALITIONS TO CATALYZE ELDER MISTREATMENT RESEARCH

**DOI:** 10.1093/geroni/igad104.0420

**Published:** 2023-12-21

**Authors:** E-Shien Chang, Sara Hackett

## Abstract

Elder mistreatment is a widespread public health problem, affecting 1 in 6 older persons every year. Despite its high frequency and severity, relatively little attention has been paid to ensuring that the findings of elder mistreatment research can be implemented in routine care and clinical practice. Forging meaningful partnerships with health care systems may hold promise to facilitate this translational process, and ultimately improve health outcomes for older persons. In this symposium, we will identify proven approaches and mechanisms to successfully build, maintain, and sustain elder mistreatment research-to-practice partnerships with various health care system stakeholders. Dr. Karl Pillemer will describe best approaches in forging researcher-practitioner collaboration with long-term care providers to implement and disseminate evidence-based resident-to-resident aggression intervention program. Dr. Lena Makaroun will examine lessons learned from four high-impact Veterans Health Administration-led collaborations to accelerate applied elder mistreatment research. Dr. Kristen Lees Haggerty will discuss strategies and successes in developing a novel elder mistreatment screening and response tool for emergency departments nationwide in partnership with the National Collaboratory to Address Elder Mistreatment. Finally, Dr. Zach Gassoumis will describe his experiences with conducting intervention and observational research as well as provide advice to early career scholars looking to build partnerships with health care systems. Taken together, these presentations will shed light on the practice, policy, and research implications in strengthening health care system partnerships to closing the discovery-delivery gap.

